# Antho-RFamide effect on light production in the bioluminescent sea pen *Pennatula phosphorea* (Octocorallia, Pennatulacea)

**DOI:** 10.1242/jeb.252487

**Published:** 2026-05-13

**Authors:** Laurent Duchatelet, Patrick Flammang, Sam Dupont, Jérôme Mallefet

**Affiliations:** ^1^ Marine Biology Laboratory, Université catholique de Louvain, B-1348 Louvain-la-Neuve, Belgium; ^2^ Biology of Marine Organisms and Biomimetics Unit, Research Institute for Biosciences, University of Mons, B-7000 Mons, Belgium; ^3^ Department of Biological and Environmental Sciences, University of Gothenburg, SE-450 34 Fiskebäckskil, Sweden

**Keywords:** Bioluminescence, Neuropeptides, Neurochemical regulation, Anthozoan, Physiological control

## Abstract

Bioluminescence in anthozoans is a rapid and coordinated response that relies on nervous control, yet the neurochemical mechanisms underlying light production remain poorly understood. In the sea pen *Pennatula phosphorea*, mechanical stimulation elicits propagating waves of green light often coupled with muscular contraction, suggesting tight integration between neural, muscular and luminous systems. Here, we investigated the presence and role of RFamide neuropeptides in the control of bioluminescence in *P. phosphorea* by combining transcriptomic analysis and pharmacological experiments. We identified Antho-RFamide-like precursor sequences in the *P. phosphorea* transcriptome, characterized by repeated conserved RFamide motifs typical of anthozoan neuropeptide precursors. Phylogenetic analysis revealed a clear differentiation between octocorallian and hexacorallian Antho-RFamide precursor sequences, while highlighting substantial variation in motif repetition number across anthozoan species. Pharmacological assays demonstrated that Antho-RFamide can trigger light emission, providing direct evidence for its involvement in luminescence control. These results support a model in which Antho-RFamide acts as an ancestral neuropeptidergic component of the bioluminescence response, operating alongside catecholaminergic pathways to regulate light emission. We further propose that variation in Antho-RFamide precursor architecture may influence neuropeptide signaling capacity and contribute to functional diversification of the neuropeptide role, including luminescence control within luminous anthozoans. By providing the first functional evidence linking neuropeptide signaling to light production in a sea pen species, this study identified a previously unrecognized role of RFamide peptides in the control of bioluminescence and revealed a complex, multi-layered neurochemical regulatory system underlying light emission in anthozoans.

## INTRODUCTION

Bioluminescence, the ability of living organisms to produce visible light through biochemical reactions, is a widespread and ecologically significant trait in marine ecosystems (Haddock et al., 2010; Martini and Haddock, 2017; Duchatelet and Dupont, 2025). Light emission generally results from the oxidation of a luciferin substrate catalyzed by a luciferase enzyme and may involve additional molecular components such as luciferin-binding proteins, fluorescent proteins and inorganic cofactors, notably calcium ions (Shimomura and Yampolsky, 2019; Delroisse et al., 2021; Duchatelet and Dupont, 2025). Although bioluminescence occurs across a broad range of marine taxa, from bacteria to vertebrates, only a limited number of systems have been comprehensively characterized at the molecular, physiological and functional levels (Lau and Oakley, 2021; Duchatelet and Dupont, 2025).

Within Cnidaria, bioluminescence is particularly well represented in Anthozoa, where luminous species are distributed across several orders, including Actiniaria, Zoantharia and Pennatulacea (Bessho-Uehara et al., 2020; DeLeo et al., 2024; Duchatelet et al., 2025; Kise et al., 2025). Pennatulaceans (sea pens) constitute the most extensively studied group of luminous anthozoans, largely because of the historical model provided by sea pansies of the genus *Renilla* (e.g. Cormier, 1960; Inoue et al., 1977; Matthews et al., 1977; Loening et al., 2007; Schenkmayerova et al., 2023). In these organisms, bioluminescence relies on a coelenterazine-dependent luciferase (*R*Luc-type), often associated with coelenterazine-binding proteins and green fluorescent proteins that modulate light emission through bioluminescence resonance energy transfer (e.g. Ward and Cormier, 1979; Kumar et al., 1990; Stepanyuk et al., 2009; Duchatelet et al., 2025). Accumulating molecular and biochemical evidence indicates that similar coelenterazine-based systems are widespread among pennatulaceans and may be evolutionarily conserved across distantly related metazoan lineages (Delroisse et al., 2017, 2021; Duchatelet and Dupont, 2025).

In sea pens, light production occurs in specialized cells, the photocytes, located within the endoderm of autozooid and siphonozooid polyps (e.g. Nicol, 1955; Wampler et al., 1973; Duchatelet et al., 2023). At the colony level, bioluminescence is typically expressed as waves of flashes propagating along the rachis, and pinnules when present, following mechanical or chemical stimulation, reflecting a coordinated response at the scale of the colony (e.g. Davenport and Nicol, 1956; Nicol, 1958; Buck, 1973; Duchatelet et al., 2023). Although the precise ecological functions of sea pen bioluminescence remain experimentally unresolved, proposed roles include antipredator defense through startle or misdirection effects, aposematic signaling or a burglar alarm mechanism that attracts secondary predators (Morin, 1976; Haddock et al., 2010; Duchatelet and Dupont, 2025).

The common sea pen, *Pennatula phosphorea*, is a shallow-water pennatulacean widely distributed in the North Atlantic and Mediterranean Sea, inhabiting soft-sediment environments at depths typically ranging from 10 to 100 m. This species is characterized by a robust axial rod anchored in the sediment and a rachis bearing pinnules densely populated with autozooid polyps. Upon mechanical stimulation, *P. phosphorea* exhibits an immediate behavioral response characterized by the retraction of polyps, accompanied by the emission of intense green bioluminescent flashes (λ_max_ ±510 nm) that propagate rapidly along the colony (Duchatelet et al., 2023, 2025).

At the biochemical level, recent studies have demonstrated that the bioluminescence of *P. phosphorea* is based on a coelenterazine-dependent luciferase system closely related to that of *Renilla*, with the additional involvement of coelenterazine-binding proteins, green fluorescent proteins and calcium ions (Duchatelet et al., 2025). Transcriptomic, immunological and biochemical approaches have confirmed the presence and expression of these molecular components within luminous tissues, supporting a conserved anthozoan bioluminescence model (Duchatelet et al., 2025).

Beyond its biochemical complexity, bioluminescence in sea pens is tightly regulated by nervous signaling. In *P. phosphorea*, as in other pennatulaceans, bioluminescence is initiated through nervous pathways, as evidenced by the rapidity of the response and its sensitivity to pharmacological manipulation (Duchatelet et al., 2023). Adrenaline acts as the principal neuroeffector triggering clusters of bioluminescent flashes, while noradrenaline and octopamine are also capable of eliciting light production (Duchatelet et al., 2023). This pattern of control is consistent with an adrenergic nervous regulation system that appears conserved across anthozoans and may derive from ancestral catecholaminergic mechanisms originally associated with neuromuscular regulation (Anctil, 1989; Pani and Anctil, 1994; Anctil et al., 2002; Kass-Simon and Pierobon, 2007).

In anthozoans, however, neuromuscular activity is rarely governed by classical neurotransmitters alone (Kass-Simon and Pierobon, 2007). A growing body of evidence indicates that muscle contraction, polyp retraction and colony-level coordination in cnidarians rely on the combined action of fast-acting neurotransmitters and slower, modulatory neuropeptide signaling (McFarlane et al., 1987; Kass-Simon and Pierobon, 2007). In models such as *Hydra* and *Nematostella*, numerous neuropeptide families, including FMFRamide-like peptides, GLWamides and lineage-specific peptides such as RPamides, have been identified and shown to regulate a broad range of functions, including muscle contraction, neuronal differentiation and developmental processes (e.g. Grimmelikhuijzen et al., 1996; Marlow et al., 2013; Takahashi and Takeda, 2015; Attenborough et al., 2019; Hayakawa et al., 2019; Zang and Nakanishi, 2020; Hauser et al., 2022). High-resolution peptidomics and receptor deorphanization studies in *Nematostella vectensis* further reveal that cnidarian peptidergic networks are extensively and evolutionarily distinct from those of bilaterians, with many G protein-coupled receptors (GPCRs) matching specific cnidarian neuropeptides (Thiel et al., 2024). This work underscores the complexity of neuropeptide signaling in cnidarians and suggests that peptidergic modulation could play roles in behaviors that require coordinated physiological responses (Thiel et al., 2024). Neuropeptides are abundantly expressed in anthozoan nerve nets and are known to regulate both excitatory and inhibitory pathways controlling contractile tissues, often acting in concert with biogenic amines (McFarlane et al., 1987; Anctil, 1989; Gallo et al., 2016). This dual mode of regulation suggests that catecholaminergic control of bioluminescence in sea pens may represent only the primary triggering component of a broader neurochemical framework.

Within this context, neuropeptides emerge as plausible candidates for modulating bioluminescent responses downstream or in parallel with catecholaminergic activation. By analogy with their established roles in anthozoan neuromuscular modulation, neuropeptides could influence the intensity, duration or spatial coordination of light emission by affecting photocyte excitability, intracellular calcium dynamics, or the coupling between nervous and contractile elements.

Based on these observations, we hypothesized that Antho-RFamide, a neuropeptide specific to the anthozoan lineage, may act as a trigger or modulator of light emission in the sea pen *P. phosphorea*. In this study, we combined transcriptomic analysis and pharmacological experiments to investigate the role of Antho-RFamide in the control of bioluminescence in this species.

## MATERIALS AND METHODS

### Transcriptome screening and Antho-RFamide sequence characterization

To identify sequences coding for Antho-RFamide precursor protein, candidate transcripts coding for anthozoan Antho-RFamide precursor proteins were retrieved based on literature data and NCBI database annotations. These potential sequences were searched in the reference *P. phosphorea* transcriptome (NCBI SRA PRJNA1152785; Duchatelet et al., 2025) using the BLAST tool kit (1 hit, *E*-value<1×10^−20^) in BioEdit software following the methodological design developed in Delroisse et al. (2018). Retrieved unigenes were then confirmed through individual searches against the NCBI NR database using tBLASTn (1 hit, *E*-value<1×10^−20^). *In silico* translation was performed on the retrieved transcript sequences using the ExPASy translate tool (https://web.expasy.org/translate/). Sequence alignments enabled the identification of Antho-RFamide specific repeated RF amide motif sequences, characteristic of anthozoan neuropeptide precursors.

The anthozoan Antho-RFamide precursor protein sequences were included in a phylogenetic analysis. Predicted protein sequences from *P. phosphorea* were aligned with Antho-RFamide precursor sequences from NCBI for *Renilla koellikeri*, *Calliactis parasitica*, *Nematostella vectensis*, *Dendronephthya gigantea*, *Paramuricea clavata*, *Exaiptasia diaphana*, *Fimbriaphyllia ancora*, *Anthopleura elegantissima* and *Actinia tenebrosa* using UGENE software (Unipro UGENE v53.0). Poorly aligned regions and regions with excessive gaps were manually removed. Phylogenetic relationships were inferred using a maximum-likelihood approach under the Jones–Taylor–Thornton (JTT) substitution model with gamma-distributed rate variation among sites, based on the trimmed alignment of Antho-RFamide precursor sequences. Because of the absence of an appropriate outgroup, phylogenetic trees were analyzed as unrooted. Given the repetitive and low-complexity nature of neuropeptide precursors, phylogenetic reconstruction was used here as a qualitative clustering tool rather than for strict evolutionary inference. Node support was assessed using 1000 bootstrap replicates. For visualization, trees were midpoint-rooted using the iTOL web tool.

### Animal collection and preparation

Common sea pens (*Pennatula phosphorea* Linnaeus 1758, *n*=12) were collected in July 2024 from the Gullmarsfjord, Sweden, using a small dredge with a 1 m aperture at a depth of 35–40 m. Animals were transported to the Kristineberg Marine Research Station (Fiskebäckskil, Sweden) and maintained in a tank supplied with a continuous flow of deep-sea water pumped from the adjacent fjord (salinity ±32.7 PSU; temperature ±8.9°C). A 15 cm layer of clean sediment was placed at the bottom of the tank to allow settlement of the sea pens. Prior to anesthetization, each colony was tested for light production by gently pinching the axial rachis with tweezers (visualization of light production pattern through mechanical stimulation) and photographed with a Sony αS7 II camera.

Specimens were anesthetized by immersion in a MgCl_2_ solution (183 mmol l^−1^ MgCl_2_, 9.9 mmol l^−1^ CaCl_2_, 27.7 mmol l^−1^ Na_2_SO_4_, 20 mmol l^−1^ Tris, pH 8.2) for 30 min. Pinnules were then dissected from each colony and weighed individually. Dissected pinnules were subsequently rinsed for 3 h in running deep-sea water prior to pharmacological assays to remove excess mucus that could interfere with luminescence measurements. Two pinnules were used per treatment per colony.

### Pharmacological assays

Luminescence measurements were performed using an FB12 tube luminometer (Tirtertek-Berthold, Pforzheim, Germany) calibrated using a standard 470 nm light source (Beta light, Saunders Technology, Hayes, UK). Experiments were conducted in a dark room, and light emission was recorded using a FB12-Sirius PC software (version 1; Tirtertek-Berthold). Luminescence was quantified as the total amount of light emitted over time (*L*_tot_), expressed in 10^9^ quanta and all values were standardized per unit of tissue mass (g).

To test the effect of Antho-RFamide on bioluminescence in *P. phosporea*, commercial Antho-RFamide (pGlu-Gly-Arg-Phe amide; P9799, Merck, Darmstadt, Germany) was applied to isolated pinnules. Each pinnule was placed in a luminometer tube containing 500 μl of artificial seawater (ASW; 400 mmol l^−1^ NaCl, 9.6 mmol l^−1^ KCl, 52.3 mmol l^−1^ MgCl_2_, 9.9 mmol l^−1^ CaCl_2_, 27.7 mmol l^−1^ Na_2_SO_4_, 20 mmol l^−1^ Tris, pH 8.2). Luminescence was recorded for 15 min following the addition of 500 μl of Antho-RFamide solution at various concentrations prepared in fresh ASW. Final concentrations (*n*=24 pinnules per concentration) of 10^−3^, 10^−4^, 10^−5^, 10^−6^ and 10^−7^ mol l^−1^ were tested. This range was selected to encompass concentrations previously reported to elicit physiological responses in anthozoan tissues while allowing detection of potential effects in intact preparations where peptide diffusion may be limited. RFamide neuropeptides typically induce physiological responses in cnidarian tissues within nanomolar to micromolar range (e.g. McFarlane et al., 1987; MacFarlane and Grimmelikhuijzen, 1991).

Positive controls consisted of either 500 μl of a KCl depolarizing solution (*n*=24; 400 mmol l^−1^ KCl, 52.3 mmol l^−1^ MgCl_2_, 9.9 mmol l^−1^ CaCl_2_, 27.7 mmol l^−1^ Na_2_SO_4_, 20 mmol l^−1^ Tris, pH 8.2) or 500 μl of adrenaline [(±)-Epinephrine hydrochloride, E4642, Merck) at a concentration of 10^−5^ mol l^−1^ (*n*=24), previously shown to trigger light production in *P. phosphorea* (Duchatelet et al., 2023). A negative control was performed by applying 500 μl of ASW (*n*=24).

### Statistical analysis

All statistical analyses were performed with R Studio (version 2023.03.1+446, 2022, R Studio Inc., Boston, MA, USA). Normality and homogeneity of variance were assessed using the Shapiro–Wilk test and Levene's test, respectively. As data did not meet assumptions of normality or homoscedasticity, even after log transformation, non-parametric tests were applied. Differences between two groups were assessed using Wilcoxon tests, while differences among multiple groups were evaluated using Kruskal–Wallis tests followed by pairwise Wilcoxon rank-sum tests. Differences were considered statistically significant at *P*<0.05. Data are presented as boxplots showing the median and interquartile range, with individual data points overlaid.

## RESULTS

### Antho-RFamide in *Pennatula*

**Fig. 1.**
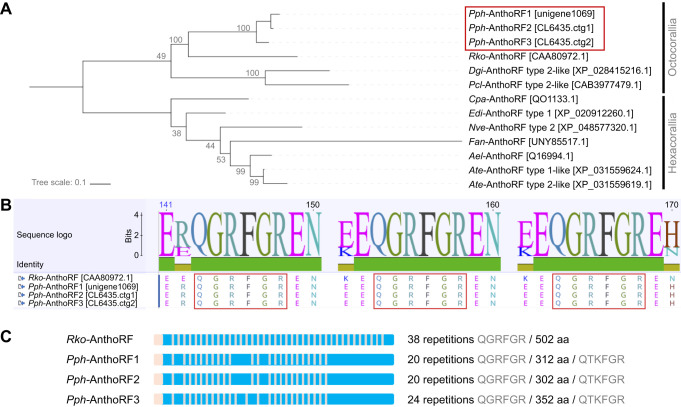
**Phylogenetic relationships, conserved motifs and repetition architecture of Antho-RFamide precursors in *Pennatula phosphorea*.** (A) Maximum-likelihood phylogenetic tree inferred from aligned Antho-RFamide precursor sequences using the JTT substitution model. Predicted *P. phosphorea* Antho-RFamide sequences (*Pph*-AnthoRF1–3; red box) form a distinct cluster closely related to the Antho-RFamide precursor of *Renilla koellikeri*. Major anthozoan clades (Octocorallia and Hexacorallia) are indicated on the right. The tree is midpoint-rooted, and branch lengths are proportional to the number of substitutions per site. (B) Sequence logo and local alignment highlighting a conserved RFamide-related motif shared between *P. phosphorea* and *R. koellikeri* Antho-RFamide precursors. Canonical QGRFGR motifs are boxed in red, illustrating strong conservation at the motif level despite divergence in the surrounding regions. (C) Schematic representation of Antho-RFamide architecture showing the distribution and number of RFamide-related motif repetitions along the sequence. *Pennatula phosphorea* Antho-RFamide precursors display fewer repetitions than those of *R. koellikeri* but retain a conserved motif core (QGRFGR), with occasional variant motifs (e.g. QTKFGR). Total sequence length and number of motif repetitions are indicated for each precursor. *Ael*, *Anthopleura elegantissima*; *Ate*, *Actinia tenebrosa*; *Cpa*, *Calliactis parasitica*; *Dgi*, *Dendronephthya gigantea*; *Edi*, *Exaiptasia diaphana*; *Fan*, *Fimbriaphyllia ancora*; *Nve*, *Nematostella vectensis*; *Pcl*, *Paramuricea clavata*; *Rko*, *Renilla koellikeri*.

The *P. phosphorea* transcriptome contained partial sequences corresponding to three predicted Antho-RFamide precursors (*Pph*-AnthoRF1–3; Fig. 1A). Predicted Antho-RFamide sequences from *P. phosphorea* form a monophyletic group, with the protein sequence from *R. koellikeri* branching as a closely related lineage, consistent with their relationship within Pennatulacea (Fig. 1A). A clear separation was observed between octocorallian and hexacorallian Antho-RFamide precursor protein sequences (Fig. 1A).

Antho-RFamide precursor sequences display a repetitive organization characterized by multiple RFamide motifs (QGRFGR) (Fig. 1B; Fig. S1). In *P. phosphorea*, predicted Antho-RFamide precursors contain several RFamide motifs distributed along the sequence, comparable to those observed in the protein from *Renilla*. Comparison of RFamide motif numbers between *P. phosphorea* and *R. koellikeri* revealed differences, with *Renilla* sequences displaying a higher number of motif repetitions than *Pennatula* (Fig. 1C).

### Antho-RFamide effects on bioluminescence

**Fig. 2.**
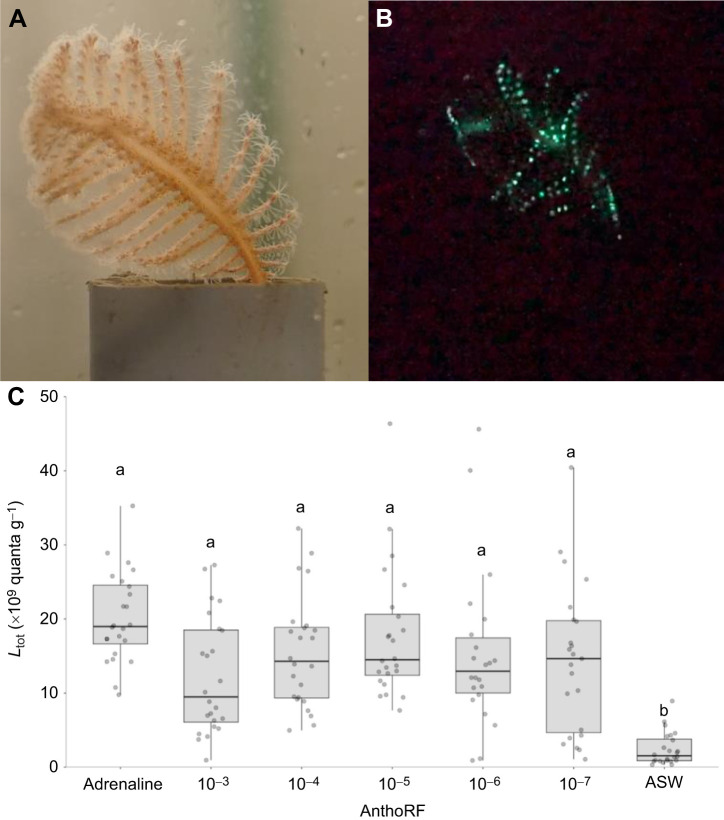
**Pharmacological effects of Antho-RFamide on the luminescence of *P. phosphorea*.** (A) A *P. phosphorea* colony under bright light prior to mechanical stimulation. (B) A propagating wave of light emitted following mechanical stimulation. (C) The total amount of light (*L*_tot_) produced by pinnules following application of Antho-RFamide at different concentrations (10^−7^–10^−3^ mol l^−1^). Adrenaline (10^−5^ mol l^−1^) and artificial sea water (ASW) served as positive and negative controls, respectively, following Duchatelet et al. (2023). All *L*_tot_ values are expressed as ×10^9^ quanta g^−1^. Different lettering indicates statistical differences (Kruskal–Wallis followed by pairwise Wilcoxon rank-sum tests).

Mechanical stimulation of *P. phosphorea* by a gentle pinching of the rachis consistently elicited waves of green light emission, indicating a mechano-nervous response propagating along the colony (Fig. 2A,B). Application of Antho-RFamide at all the tested concentrations induced bioluminescent flashes in pinnules. Flashes of light emission generally occurred over the first 3 min after Antho-RFamide application (Fig. S2). All Antho-RFamide concentrations triggered a light emission significantly higher than that measured in the ASW control (pairwise Wilcoxon test, *P*<0.05; Fig. 2C). Antho-RFamide elicited a significant response in isolated pinnules at 10^−7^ mol l^−1^. Increasing the peptide concentration up to 10^−3^ mol l^−1^ did not result in a statistically significant increase in response amplitude. Responses were similar across concentrations (pairwise Wilcoxon test, *P*>0.05; Fig. 2C), with a slight non-significant decrease at the highest concentration tested (10^−3^ mol l^−1^). These results indicate that maximal activation was already reached at the lowest concentration tested. Light emission recorded following the application of adrenaline (10^−5^ mol l^−1^) did not differ statistically from responses elicited by any of the Antho-RFamide concentrations tested (pairwise Wilcoxon test, *P*>0.05; Fig. 2C). Mean *L*_tot_ values are shown in Table S1 and indicate similar levels of light emission across all Antho-RFamide concentrations. Application of KCl induced luminescence, with a mean *L*_tot_ value of 293.57(±40.95)×10^9^ quanta g^−1^.

## DISCUSSION

The presence of Antho-RFamide-like sequences in the *P. phosphorea* transcriptome further supports the widespread occurrence of RFamide neuropeptides within Anthozoa (Koch and Grimmelikhuijzen, 2019). Three partial Antho-RFamide-like sequences were identified. Rather than reflecting true biological multiplicity, the presence of several partial transcripts probably results from redundancy or fragmentation during transcriptome assembly. Nevertheless, duplication of the sequence could not be excluded, as multiple sequences were also found in other anthozoans (Koch and Grimmelikhuijzen, 2020). The repeated occurrence of the conserved QGRFGR motif is a typical feature of RFamide neuropeptide precursors (Schmutzler et al., 1992; Reinscheid and Grimmelikhuijzen, 1994; Koch and Grimmelikhuijzen, 2019) and should therefore be interpreted as a genuine biological characteristic rather than an assembly artefact. Minor sequence variations observed among the partial transcripts can most parsimoniously be explained by assembly-related discrepancies. Nevertheless, comparative analysis revealed a clear differentiation between octocorallian and hexacorallian Antho-RFamide sequences, consistent with lineage-specific diversification while retaining a conserved anthozoan signature.

Across anthozoan species, the number of repeated RFamide motifs within Antho-RFamide precursors varies markedly. For example, the sea pansy *R. koellikeri* possesses approximately 38 copies of Antho-RFamide, whereas *A. elegantissima*, *Acanthogorgia aspera* and *C. parasitica* contain 13, 27 and 19 copies, respectively (Schmutzler et al., 1992; Reinscheid and Grimmelikhuijzen, 1994; Koch and Grimmelikhuijzen, 2019). Such variations in precursor architecture probably reflect differences in the potential output of mature RFamide peptides following precursor processing, which may influence the strength and dynamics of RFamide signaling and contribute to functional diversification within the anthozoan.

RFamide neuropeptides are well known to participate in the nervous regulation of a wide range of physiological processes in anthozoans, including muscle contraction, sensory integration and neuromodulation (Grimmelikhuijzen et al., 1986, 1996; Marlow et al., 2013; Takahashi and Takeda, 2015; Attenborough et al., 2019; Hayakawa et al., 2019; Zang and Nakanishi, 2020; Hauser et al., 2022). Neuropeptides function as both neuroeffectors and neurorepressors within a diffuse nerve net, compensating for the absence of centralized nervous structures (Grimmelikhuijzen and Westfall, 1995; Westfall et al., 2002; Kass-Simon and Pierobon, 2007). For example, Antho-RFamide induces increased muscle tone and contraction in the sea anemone *C. parasitica* at concentrations in the micromolar to sub-micromolar range (10^−6^–10^−7^ mol l^−1^) (McFarlane et al., 1987). In this context, the identification of Antho-RFamide in *P. phosphorea* is particularly relevant given the species’ rapid and coordinated bioluminescence response to mechanical stimulation (Nicol, 1955, 1958; Morin, 1976). Sea pens produce propagated waves of light following mechanical perturbation, a response that is initiated by a nervous impulse transmitted through the mesogleal nerve net, ultimately resulting in both muscular contraction and light emission at the level of the polyps (Nicol, 1955, 1958; Duchatelet et al., 2023).

Notably, both *Pennatula* and the closely related sea pansy *Renilla* possess Antho-RFamide precursors containing a relatively high number of repeated peptide motifs. While the functional significance of this feature remains unresolved, such precursor architectures are generally associated with an increased capacity for neuropeptide production and release (Grimmelikhuijzen et al., 1996; Wegener and Gorbashov, 2008; Koch and Grimmelikhuijzen, 2020). This characteristic may be compatible with the high and rapid neuropeptidergic demand required to sustain propagating luminescent waves, although direct experimental evidence is currently lacking.

Our pharmacological experiments demonstrate that Antho-RFamides trigger light production in *P. phosphorea* across all tested concentrations, providing functional evidence for the involvement of this neuropeptide in bioluminescence control. The amount of light produced following Antho-RFamide application is comparable to that elicited by adrenaline. The absence of a dose-dependent increase above 10^−7^ mol l^−1^ suggests that the response of pinnule tissues was already saturated at the lowest concentration tested and that Antho-RFamide acts within a concentration range comparable to that reported in other cnidarian systems (McFarlane et al., 1987; MacFarlane and Grimmelikhuijzen, 1991). This pattern is consistent with the high affinity typically reported for RFamide receptors in cnidarians (Gründer et al., 2023; Thiel et al., 2024). In contrast, application of potassium chloride induced a response approximately 20 times stronger. Although this treatment does not represent a physiological stimulus, KCl is known to cause non-specific membrane depolarization and massive neurotransmitter release, thereby activating the nervous circuitry underlying light emission. This marked increase in luminescence therefore confirms the nervous control of the process rather than reflecting a naturally occurring regulatory pathway. As observed in other luminous metazoans, the physiological control of bioluminescence is unlikely to rely on a single signaling molecule (e.g. De Bremaeker et al., 1996, 1999, 2000). Instead, light production appears to be modulated by multiple interacting pathways (De Bremaeker et al., 1996, 1999, 2000; Duchatelet et al., 2021; Duchatelet and Dupont, 2025). Antho-RFamide may therefore represent an ancestral and primary neuropeptidergic component involved in triggering luminescence, with catecholamines or other, as yet untested, neuromodulators acting in parallel or downstream (synergistically or antagonistically) to fine-tune the response.

Interestingly, isolated photocytes of the sea pansy *R. koellikeri* only occasionally react to Antho-RFamide, and only at high concentration (10^−3^ mol l^−1^; Germain and Anctil, 1988). This apparent low sensitivity may reflect differences in activation thresholds, a predominantly modulatory rather than directly excitatory mode of action, or the requirement for close cellular interactions within intact tissue, where Antho-RFamide signaling could act indirectly via neurons or neighboring muscular cells. Such context-dependent signaling is consistent with neuropeptide function in diffuse nerve nets, where peptide effects often rely on local concentration gradients, cellular proximity and the integration of multiple signaling pathways. Immunohistological studies performed in *Renilla* tissues highlight the important role of this system, showing the vast array of expression of Antho-RFamide-containing neurons within both autozooids and siphonozooids, from the muscular walls, mesenteric filaments, including follicles containing either ovocytes or spermatophores, endodermal channels connecting the various colonial compartments, and the circular muscle of the peduncle (Pernet et al., 2004).

A similarly high level of regulatory complexity is found in the control of bioluminescence in the brittle star *Amphipholis squamata*. Indeed, multiple neuroeffectors and neuromodulators, including neuropeptides, have been shown to regulate light emission in this species. Bioluminescence is primarily triggered through a cholinergic pathway, with acetylcholine identified as the main neuroeffector directly inducing luminescence (De Bremaeker et al., 1996, 2000). However, other classical neurotransmitters, including glutamate and purines, are also capable of eliciting light production (De Bremaeker et al., 1996, 2000). Beyond direct excitation, several signaling molecules modulate the intensity and duration of the luminescence response. These include adrenaline, γ-aminobutyric acid (GABA), glycine and the echinoderm-specific neuropeptide SALFamide S1, which potentiates acetylcholine-induced luminescence (De Bremaeker et al., 1999, 2000; Dupont et al., 2004). The presence of SALFamide peptides within ophiuroid tissues further confirms their physiological relevance. Conversely, serotonin, noradrenaline and dopamine act as neurorepressors, inhibiting light emission (De Bremaeker et al., 2000). Together, these findings demonstrate that bioluminescence in this deuterostome invertebrate is governed by a multilayered neurochemical system in which classical neurotransmitters initiate light production, while neuromodulators and neuropeptides fine-tune its amplitude and temporal dynamics, integrating excitation, modulation and inhibition to achieve precise control of light output (De Bremaeker et al., 1999).

Overall, our results support a model in which Antho-RFamide neuropeptides play at least an effector role in the nervous control of bioluminescence in *P. phosphorea*, acting within a broader network of neuromodulatory signals, including catecholaminergic pathways. Despite these advances, the precise cellular and anatomical localization of Antho-RFamide peptides and their cognate GPCRs in *P. phosphorea* remains unknown. Further investigations are required to determine whether Antho-RFamide signaling acts directly on luminous cells, indirectly via muscular or neuronal intermediates, or through a combination of the two. Notably, luminous cells expressing the *P. phosphorea* luciferase (*Pph*-Luc) are located in close proximity to muscular cells (Duchatelet et al., 2025), suggesting a tight functional coupling between contraction and light emission. Both processes are known to rely on calcium as a key second messenger, raising the possibility that Antho-RFamide-induced calcium signaling would constitute a shared regulatory pathway linking muscular contraction and bioluminescence. Such coupling would be consistent with the rapid and coordinated nature of the light response observed following mechanical stimulation.

This study provides the first functional evidence that RFamide neuropeptides are directly involved in the control of bioluminescence in an anthozoan, moving beyond their previously inferred or descriptive roles. By combining transcriptomic identification with pharmacological validation, we demonstrate that Antho-RFamide is not only present but actively contributes to light production in *P. phosphorea*. These findings support the existence of a multi-layered neurochemical regulatory system, in which neuropeptidergic signaling operates alongside classical neurotransmitters to control light emission. More broadly, our results suggest that such integrated neurochemical control may represent an evolutionarily conserved feature of luminous systems, linking neuromuscular coordination and light production. This work, therefore, identifies a new functional role of RFamide neuropeptides in anthozoans and provides a framework for future studies to investigate the cellular targets and evolutionary origins of bioluminescence control, notably emphasizing the need for integrative approaches that combine omics, pharmacology and cellular localization to fully elucidate the mechanisms underlying light production in anthozoans.

## Supplementary Material

10.1242/jexbio.252487_sup1 Supplementary information
