# Supplementary material for: Antho-RFamide effect on light production in the bioluminescent sea pen Pennatula phosphorea (Octocorallia, Pennatulacea)
Source: J Exp Biol. 2026 May 13;229(9):jeb252487. doi: 10.1242/jeb.252487 (PMC13245900; doi:10.1242/jeb.252487)
Supplement: Supplementary information [file jexbio-229-252487-s1.pdf]

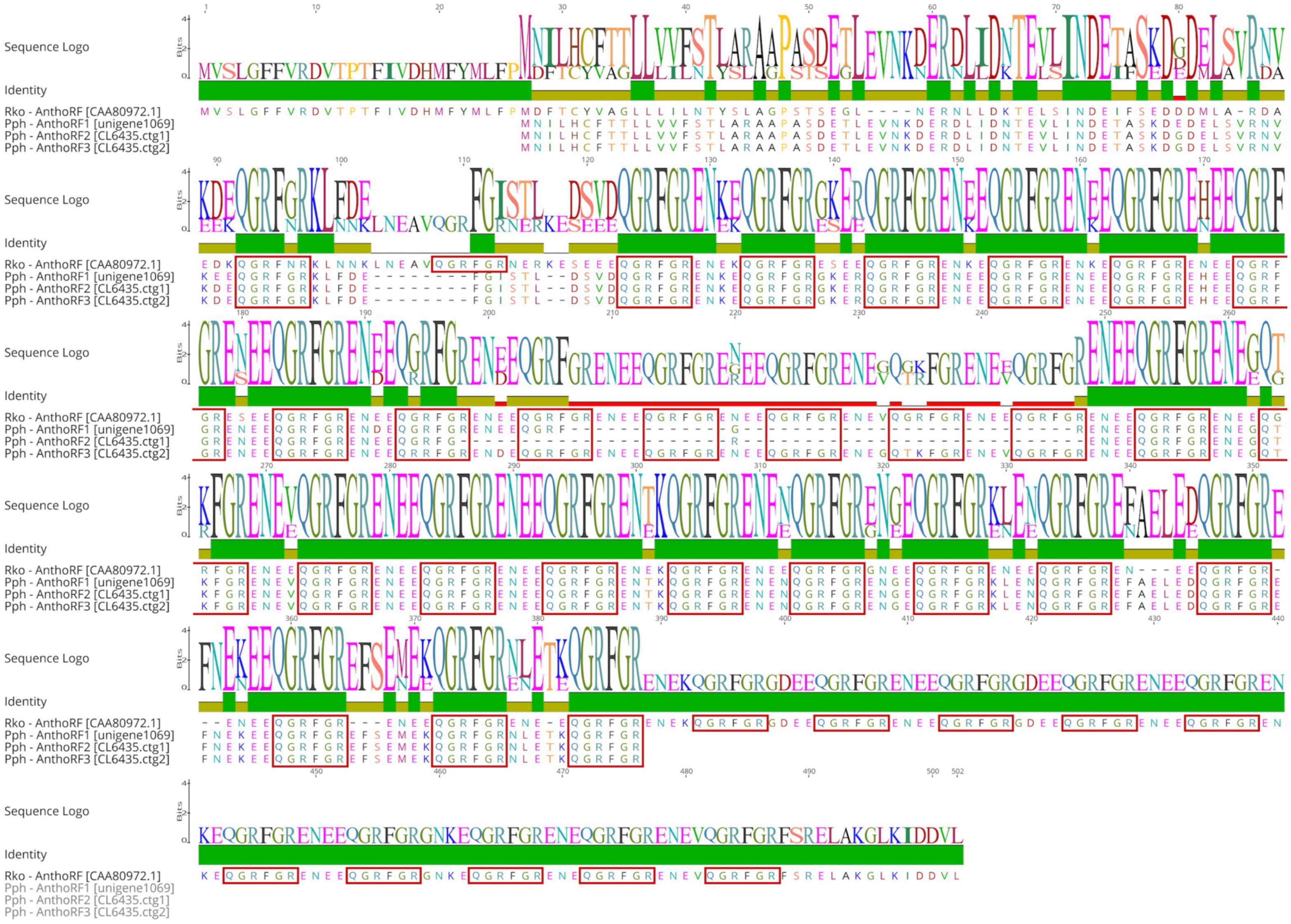

Fig. S1. Full-length alignments of *Renilla koellikeri* Antho-RFamide precursors with the three retrieved partial sequences of *Pennatula phosphorea Antho*-RFamide precursors. Red boxes underline the repeated QGRFGR motifs or the variant QTKFGR, shared between sequences.

Journal of Experimental Biology • Supplementary information

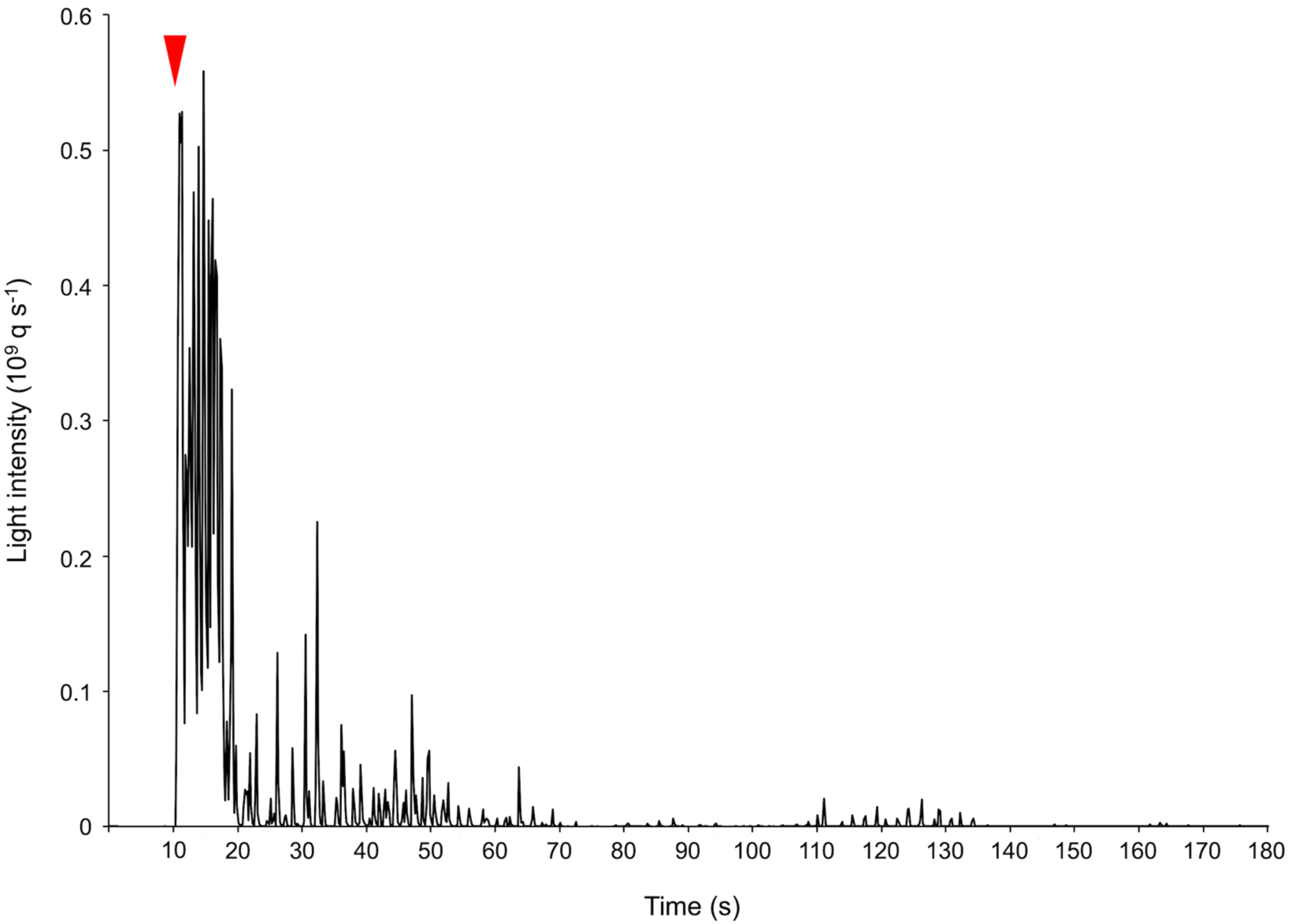

Fig. S2. Original recording of Antho-RFamide 10^{-5} mol l^{-1} application on pinnules over the first three minutes of an assay. Arrowhead indicates the Antho-RFamide application.

Journal of Experimental Biology • Supplementary information

Table S1. Mean Ltot values recorded for each treatment during the Antho-RFamide pharmacological assay (n = 24 for each treatment).

**Treatment**	**Ltot (Mean ± S.E.M) (10^9 q g^{-1})**
ASW	2.42 ± 0.45
Adrenaline (10^{-5} mol l^{-1})	20.19 ± 1.24
Antho-RFamide (10^{-7} mol l^{-1})	16.2 ± 2.69
Antho-RFamide (10^{-6} mol l^{-1})	19.15 ± 3.47
Antho-RFamide (10^{-5} mol l^{-1})	17.71 ± 1.81
Antho-RFamide (10^{-4} mol l^{-1})	15.47 ± 1.54
Antho-RFamide (10^{-3} mol l^{-1})	12.20 ± 1.60

Journal of Experimental Biology • Supplementary information
